# Impact of Ultrasound Treatment and pH-Shifting on Physicochemical Properties of Protein-Enriched Barley Fraction and Barley Protein Isolate

**DOI:** 10.3390/foods9081055

**Published:** 2020-08-04

**Authors:** Pia Silventoinen, Nesli Sozer

**Affiliations:** VTT Technical Research Centre of Finland Ltd., P.O. Box 1000, FI-02044 VTT, Finland; nesli.sozer@vtt.fi

**Keywords:** protein-enriched barley fraction, barley protein isolate, ultrasound, colloidal stability, protein solubility, particle size

## Abstract

Ultrasonication alone or in combination with a pH-shifting method could be applied as means for improving the techno-functional properties and performance of barley protein ingredients in liquid food matrix. Ultrasound technology was utilised with and without pH-shifting to 3, 7 and 9 aiming at investigating their impact on primary protein structure, protein solubility, particle size and colloidal stability of an air-classified protein-enriched barley fraction and a barley protein isolate. Shifting the pH of sample dispersion to 9 followed by neutralisation to pH 7 improved protein solubility and colloidal stability of the isolate whereas it had less impact on the protein-enriched fraction. Ultrasound treatment improved both protein solubility and colloidal stability of the protein-enriched fraction at alkaline pH and particle size reduction by ultrasonication was observed at all the studied pH-values. For protein isolate, ultrasonication improved protein solubility at all pH-values and colloidal stability was improved at acidic and neutral pH whereas the sample was inherently stable at alkaline pH. The protein profiles of both ingredients remained unaffected by ultrasound treatment. The results suggest adopting ultrasonication as a promising tool for improving applicability of barley protein ingredients in liquid food systems.

## 1. Introduction

Barley is a globally important crop, ranking fourth in production among cereals [1]. However, food use of barley ingredients, especially barley protein, is limited, as barley is mainly utilised for brewing or as feed. Considering the acute global challenges such as population growth and climate change, it is necessary to develop alternatives to animal-based proteins [2]. Plant-based proteins and ingredients provide a more sustainable option for food ingredients. However, plant proteins are known to exhibit poor techno-functional properties, which hinder their applicability in food products [3]. The main class of barley proteins is prolamins (35–55%), which are soluble in aqueous alcohol and the second most abundant protein class is glutelin being soluble in acid/base [4,5]. Food use of barley proteins is restricted by their low solubility in water (9–29%) at food relevant pH ranging from 3 to 9 [5,6,7,8]. However, there is limited literature available on improving solubility or other techno-functional properties of barley proteins [9,10].

The techno-functional properties of plant proteins can be modified by physical, chemical and bioprocessing. Ultrasound technology can be employed to alter properties of food components in liquid media. The high intensity, low frequency (16–100 kHz) ultrasound treatment exploits mechanical acoustic waves that generate oscillating compression and rarefaction cycles in the liquid media. During these compression cycles, small gas bubbles originally present in the liquid media grow, eventually resulting in critical size and collapse, i.e., cavitation [11,12,13]. Cavitation causes accumulation of energy locally when rapid and short heating up to 5500 °C and increases in pressure up to 50 MPa, occur [13]. In addition, cavitation induces other phenomena, such as mixing, microstreaming currents, shear stresses, and turbulence [11,12,14] that contribute to altering the properties of the treated media. Ultrasound technology has relatively low energy consumption and environmental impact, which makes it a green food processing technology. Regarding plant protein functionalisation, ultrasound treatment has improved water solubility of soy [15,16,17,18,19], pea [20], black bean [21], millet [22], faba bean [23] and canola [24] proteins. It has also decreased particle size of several pulse proteins [16,17,20,23,25,26]. Additionally, ultrasonication has, for example, modified surface hydrophobicity of soy [16,19] and pea protein isolates [20,26], zeta-potential of black bean protein isolate and millet protein concentrate [21,22], improved foaming properties of canola and pea protein isolates [24,26], as well as oil absorption capacity, emulsifying, and gelation properties of canola protein isolate [24]. However, applicability of ultrasound for cereal protein ingredient functionalisation remains less studied and most of the literature considering ultrasound treatment of cereal proteins has focused on hydrolysis of rice protein [27,28,29]. No scientific report to date has been documented on the effects of ultrasound treatment on barley protein functionality.

In addition to physical treatments, the pH-shifting method may result in structural protein modifications. In this method, the proteins are exposed to extreme alkaline or acidic conditions for a certain time followed by pH adjustment to neutral. The modifications based on pH-shifting are reported to be due to changes in tertiary structure, e.g., partial unfolding and refolding, while secondary structure remains unaffected [30]. The phenomena can also be referred to as formation of a molten globule state [31,32]. For plant proteins, research regarding pH-shifting has concentrated more on soy and less on other plant proteins. Surface hydrophobicity, emulsifying properties [33] and protein solubility [34] of soy protein isolate improved after pH-shifting to highly acidic and alkaline conditions. For pea protein isolate, pH-shifting to 12 improved protein solubility and increased free sulfhydryl group content [20]. Combination of pH-shifting and ultrasound treatment has not been reported for cereal protein functionalization.

We have previously reported an air classification-based production of a protein-enriched barley fraction [7]. The fraction had limited functional properties, such as low protein solubility. This work aimed at improving the techno-functional properties of the protein-enriched barley fraction using ultrasound technology and/or pH-shifting approach. In addition to protein-enriched barley fraction, a barley protein isolate was studied. Among the various techno-functional properties emphases was given to protein solubility and colloidal stability, as they are essential quality predictors for liquid food applications. The protein-enriched barley fraction and barley protein isolate were compared in terms of the impact of ultrasound treatment and pH-shifting on the physicochemical properties.

## 2. Materials and Methods

### 2.1. Raw Material

Industrial barley endosperm fraction was obtained from Altia Corporation (Koskenkorva, Finland) and dry-processed to obtain barley grits with 27% mass yield as described in Silventoinen et al. [7]. The grits were pin disc-milled and sieved with 150 µm sieve resulting in fractions <150 µm (40% mass yield) and >150 µm (60% mass yield). The fraction <150 µm, i.e., the barley endosperm fraction, accounted for 11% of the barley grain, contained 8.3% protein and was utilized as a raw material in this work.

### 2.2. Preparation of Protein-Enriched Barley Fraction

Protein-enriched barley fraction (PEBF) was prepared by mixing the industrial barley endosperm fraction with 0.5% (*w/w*) flow aid (Aerosil 200F) and air classifying the sample in industrial scale using a Hosokawa Alpine 200 ATP St. eng air classifier (Hosokawa Alpine, Augsburg, Germany). The air classifier was operated with speed of 4800 rpm using central and tangential air flows of 500 and 450 m^3^/h, respectively, and an average feed rate/throughput of 189 kg/h. Air classification resulted in a fine protein-enriched fraction with mass yield of 14% from the raw material, calculated as described in Silventoinen et al. [7].

### 2.3. Preparation of Protein Isolate

Barley protein isolate (BPI) was prepared from the air-classified protein-enriched barley fraction (PEBF) using alkaline extraction and isoelectric precipitation according to a method described in Wang et al. [5]. In brief, PEBF was dispersed in Milli-Q water (10% *w/w*), and mixed for 1 h. The pH of the suspension was adjusted to 11 (using 1M NaOH). The dispersion was mixed for 30 min and centrifuged (10000× *g*, 15 min, 20 °C). The supernatant was collected and the pH was reduced to 5 (using 1M HCl) followed by 30 min stirring and centrifugation (10000× *g*, 15 min, 20 °C) in order to separate the precipitated proteins. The solid residue, i.e., the protein isolate, was freeze dried and milled with a laboratory scale ultra-centrifugal mill ZM200 equipped with a 0.5 mm sieve (Retsch, Hann, Germany).

### 2.4. Biochemical Analysis

Protein content was determined based on total nitrogen content (N x 6.25) analyzed by Kjeldahl method according to the AOAC method 2001.11 using an autoanalyser (Foss Tecator Ab, Höganäs, Sweden) [35]. Starch content was analysed according to the AACC method 76–13.01 using a total starch assay kit (Megazyme, Bray, Ireland). All biochemical analysis were performed as duplicates.

### 2.5. Ultrasound Treatment

**Figure 1:**
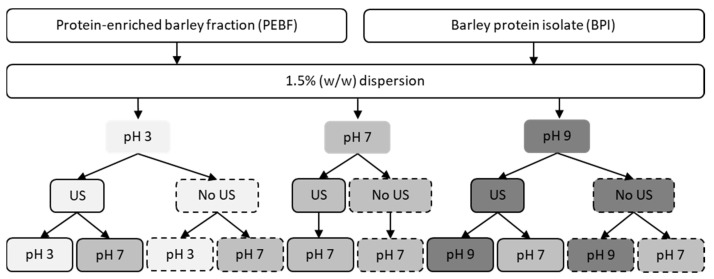
Schematic presentation of ultrasound and pH-shifting treatments performed for the protein-enriched barley fraction (PEBF) and barley protein isolate (BPI). US; ultrasound treatment.

The procedure for ultrasound treatment and pH-shifting was adopted from Jiang et al. [20] with modifications, and is illustrated in Figure 1. 

PEBF and BPI dispersions (1.5% *w/w*) were prepared by magnetic stirring of the powder samples in Milli-Q water for 30 min followed by pH adjustment to 3, 7 or 9 (using 1M HCl or NaOH). The samples were stirred for 30 min further and pH was readjusted if necessary. Control samples were kept under constant magnetic stirring at room temperature whereas ultrasound-treated samples were processed with ultrasound using a VC 750 ultrasonic processor (Sonics & Materials, Inc., Newtown, CT, USA). The processor was equipped with 13 mm diameter stainless steel probe and operated at 20 kHz using an amplitude of 100%. Ultrasound treatment was performed for 2.5 min followed by cooling and another 2.5 min ultrasound treatment. The treatment was performed for 75 mL of dispersion placed in a 150 mL flat-bottomed cylinder (diameter 54 mm), which was immersed in an iced water bath to prevent samples from overheating. The ultrasound probe was placed 0.5 cm up from the bottom of the treatment cylinder. The temperature of the dispersions was measured to be <35 °C during the treatments. After the ultrasound treatment, the dispersions were left at room temperature for 60 min (magnetic stirring) and the pH was readjusted to 7 in aliquots (magnetic stirring for 30 min and readjustment if needed) whereas other aliquots remained at pH 3 or 9. The samples were used as fresh directly after the treatments, unless otherwise stated. Ultrasound treatments were performed at least in duplicate.

### 2.6. Physicochemical Characteristics

#### 2.6.1. Protein Composition

Molecular weight distribution of the proteins in PEBF and BPI was visualized by sodium dodecyl sulphate-polyacrylamide gel electrophoresis (SDS-PAGE) [36] under reducing and non-reducing conditions. The analysis under reducing conditions was performed as described in Silventoinen et al. [37]. The analysis under non-reducing conditions was carried out otherwise similarly but no β-mercaptoethanol was added to the sample buffer. The protein bands were visualised with Criterion Stain Free Imager and examined using Image Lab software (Bio-Rad Laboratories Inc., Hercules, CA, USA).

#### 2.6.2. Protein Solubility

Ultrasound-treated and control samples were centrifuged at 10000× *g* for 10 min at 20 °C. The amount of soluble protein was quantified from the supernatants using a commercial kit (DC Protein Assay, Bio-Rad), which is based on the Lowry protein assay [38]. Bovine serum albumin (Sigma-Aldrich, St. Louis, MO, USA) was used as a standard protein. Protein solubility (PS) was calculated as follows:

PS (%) = c_s_/c_i_ * 100%, (1)

where c_s_ is the protein concentration in the supernatant (mg/mL) and c_i_ is the protein concentration in the initial suspension (mg/mL).

#### 2.6.3. Particle Size

The particle size distribution of the dispersions was analysed by laser diffraction with Beckman Coulter LS 230 (Beckman Coulter Inc., Brea, CA, USA) using filtered Milli-Q water as dispersant. The refractive indices of 1.33 and 1.50 were used for the dispersant and the particles, respectively. The volume-weighted mean particle diameter was analysed in duplicate measurements from duplicate treatments (*n* = 4).

#### 2.6.4. Colloidal Stability

Colloidal stability of samples (8 mL) was evaluated by comparison of their photos, which were taken 10 and 30 min after mixing. Photos are representative of duplicate analysis.

### 2.7. Statistical Analysis

Statistical analysis of the protein solubility values was performed using SPSS Statistics software (version 24, IBM, Armonk, NY, USA). 6–10 replicate results from each experiment (two replicate treatments) were analysed by one-way analysis of variance (ANOVA). The level of significance was set at *p* < 0.05 and was assessed by Tukey’s post hoc test.

## 3. Results and Discussion

### 3.1. Protein Fractionation and Isolation

Air classification of the barley endosperm fraction resulted in protein enrichment from 8.3% in the raw material (data not shown) to 24.0% in the protein-enriched barley fraction (PEBF) (Table 1). Starch content was decreased (data not shown) and the fine PEBF from air classification contained 55.6% starch. During alkaline extraction of barley protein from the PEBF, protein content increased from 24.0 to 85.8% in barley protein isolate (PBI) and mass and protein yields in the extraction were 20.1 and 72.0%. Wang et al. [5] reported similar values during alkaline extraction of barley proteins from pearled barley grain flour. As expected, starch content decreased clearly from 55.6 to 0.6% during wet extraction of proteins.

### 3.2. Impact of Ultrasound Treatment and pH-Shifting on Protein Profile

**Figure 2:**
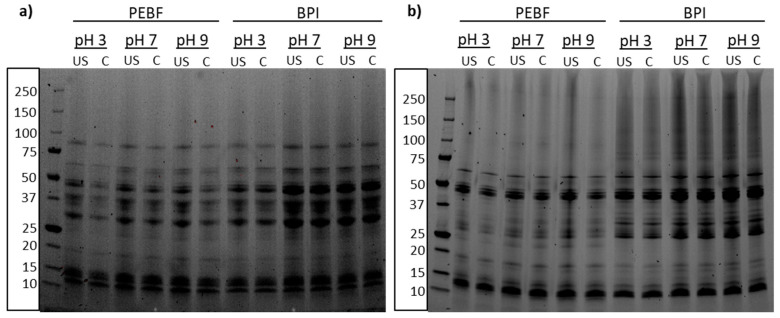
SDS-PAGE of protein-enriched barley fraction (PEBF) and barley protein isolate (BPI) at different pH-values without (C) or after ultrasound-treatment (US) analysed under (**a**) reducing and (**b**) non-reducing conditions.

SDS-PAGE analysis of PEBF and BPI revealed modest changes in the protein profiles of the two ingredients (Figure 2a, control samples at pH 7). B and C hordeins form the most abundant protein classes in barley and were identified at 30–50 kDa [5,39] and 55–70 kDa [40], respectively, in both PEBF and BPI. However, slightly more intense bands were observed from BPI suggesting enrichment of those proteins into BPI during wet extraction of proteins. In addition to B and C hordeins, presence of D hordeins at approximately 85–90 kDa and low molecular weight or A hordeins at below 15 kDa, were identified [5,39]. Gamma globulin subunits are known to size 50, 40, 25 and 20 kDa [41] and those subunits were detected in both samples (Figure 2a). Non-reducing SDS-PAGE revealed some differences between PEBF and BPI as the polypeptide at around 25 kDa was not present in BPI whereas larger polypeptides at 75–150 kDa were more abundant in BPI than in PEBF.

The clear bands in the protein profiles were unaffected by the ultrasound treatment, both under reducing and non-reducing conditions, whereas slightly more smearing was observed throughout the whole molecular weight distribution for the ultrasound-treated than for the control samples. Presence of all the same protein bands in both control and ultrasound-treated samples prove that no protein degradation or aggregation was observed as a result of ultrasound treatment. Thus, no changes in the primary protein structure took place during ultrasonication. In accordance with the current results, no changes in the primary structure due to ultrasound treatment have been observed for example for pea [42], soy [19], canola [24], and rice protein isolates [25]. On the other hand, Nazari et al. [22] and Resendiz-Vazquez et al. [43] reported decreased molecular weight as a result of ultrasound treatment of millet protein concentrate and jackfruit seed protein isolate, respectively. Thus, the impact on protein structure is varying depending on the ultrasonic intensity applied and the plant matrix.

The smearing in the ultrasound-treated samples was also noted by Jiang et al. [20], who observed creation of wider bands from the ultrasound-treated samples and attributed it to increased surface hydrophobicity, which has an effect on the material behaviour on the SDS-PAGE gel. Furthermore, slightly stronger smearing was observed for the BPI than PEBF under non-reducing conditions which may be attributed to partial protein denaturation during the alkaline extraction, as discussed by Rommi et al. [44] for rapeseed press cake proteins.

### 3.3. Protein Solubility

**Figure 3:**
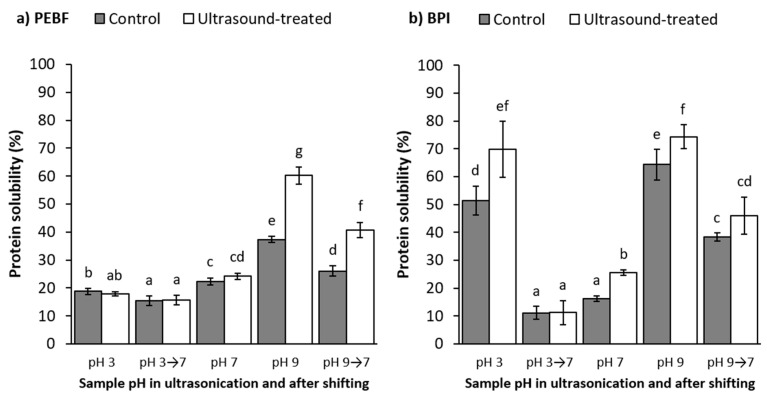
Impact of ultrasound treatment and pH on protein solubility of (**a**) protein-enriched barley fraction (PEBF) and (**b**) barley protein isolate (BPI). Shifting the sample pH to 7 after ultrasound treatment is indicated with an arrow. Values represent means and error bars standard deviations of 2–4 parallel protein analysis performed after each of the two replicate extractions (*N* = 6–10). Results with different letters, separately for the two raw materials, are significantly (*p* <0.05) different from each other.

The amount of soluble protein in aqueous dispersions of PEBF and BPI in their native pH conditions (pH 5.9 for PEBF and 5.0 for BPI) was 14.7 and 2.9%, respectively. Isoelectric point of barley proteins is between pH 5 and pH 6 [5,6,9], which supports the relatively low protein solubility values obtained at that pH-range. Shifting the pH towards acidic (pH 3), neutral (pH 7) or alkaline (pH 9) increased the protein solubility of PEBF to 18.8, 22.4 and 37.3%, respectively (Figure 3a, control samples). In BPI, the solubility increase was more pronounced at acidic (51.4%) and alkaline (64.3%) conditions, whereas it remained moderate at pH 7 (16.1%) (Figure 3b, control samples). This resulted in clearly a U-shaped solubility curve, similar to earlier reports for barley proteins [6,9]. The difference in the protein solubility between PEBF and BPI, especially at acidic and alkaline conditions, most probably resulted from the subtle differences in protein compositions due to production processes of these ingredients, as observed also in SDS-PAGE (Figure 2). Similar differences between solubility of proteins in barley flour and barley protein isolate have been reported by Yalçin and Çelik [8]. Moreover, the high starch content in PEBF may have affected the protein solubility analysis, which requires further investigation.

#### 3.3.1. Effect of pH-Shifting

Shifting of the dispersion pH to 9 and then re-adjusting back to pH 7 increased slightly the protein solubility of PEBF compared to the dispersion which remained at pH 7 all through the stirring duration (22.4% for control at pH 7 vs. 26.1% for control shifted to pH 9 and back to pH 7) (Figure 3a). For BPI, shifting the pH to 9 and back to 7 resulted in more pronouncedly significantly (*p* < 0.05) increased solubility (38.3%) when compared to the control at pH 7 (16.1%). Jiang et al. [20] observed that shifting the pH of pea protein isolate dispersion to pH 12 and back to pH 7 resulted in dramatic increase in protein solubility from 8.2 to 54.9%. The authors postulated that the increase in protein solubility was due to partial unfolding of the protein structure at alkaline conditions followed by refolding and formation of a molten globule state at neutral pH, increased flexibility in structure and potential increase in ionic interactions between water and charged amino acids [20,30,34]. Similarly, increase in solubility of soy protein isolate was also reported by Lee et al. [18] during pH-shifting to highly alkaline pH of 12 and readjusting to neutral. However, Jiang et al. [20] did not observe significant differences in the protein solubility of pea protein isolate when shifting to milder alkaline condition of pH 10 and back to pH 7 (9.8 vs. 8.2%) and similar observation was also noticed by Lee et al. [18]. In the current research, pH shifting to even milder alkaline conditions (pH 9) and back to pH 7 showed positive impact on protein solubility of BPI, which may be attributed with formation of the previously hypothesised molten globule state. On the other hand, no positive impact of pH-shifting to alkali (pH 9) and back to pH 7 was observed for PEBF compared to the solubility at pH 7 without pH-shifting. Differences in the solubility values of BPI and PEBF may result from the initial alkaline extraction step of BPI or the presence of greater amount of non-protein compounds in the PEBF.

On the contrary to what was observed in alkaline pH-shifting, a significant (*p* < 0.05) decrease in the protein solubility was observed for PEBF shifted to pH 3 and back to pH 7 (15.3% vs. 22.4%). Also for BPI, a slight decrease in solubility was observed (11.2% vs. 16.1% for pH-shifted and control, respectively). In compliance with these results, Jiang et al. [20] observed a slight decrease in solubility from 8.2 to 6.7% when shifting pea protein isolate to pH 4 and back to pH 7. Likewise, Lee et al. [18] did not notify any improvements in soy protein solubility as a result of acidic pH-shifting followed by neutralisation. Thus, molten globule state or similar phenomena, does not seem to apply in acidic pH-shifting of the studied plant proteins and this has been stated to apply for other plant proteins as well [30].

#### 3.3.2. Effect of Ultrasound Treatment

Ultrasound treatment almost doubled the protein solubility of PEBF (from 37.3 to 60.3%) at alkaline pH. On the other hand, no impact on the protein solubility of PEBF was observed at neutral and acidic pH. For BPI, ultrasonication significantly (*p* < 0.05) increased the protein solubility at all three pH values (Figure 3b). Similar increases in protein solubility after ultrasonication have also been observed for various plant proteins. Ultrasound treatment increased solubility of black bean protein at pH 7 from 47 to 51% [21], pea protein at pH 7 from 8 to 56% [20], millet protein at pH 7 from 67 up to 95% [22], faba bean protein at pH 7.4 from 20 to 35% [23] and soy protein at pH 8 from 10 up to 55% [19]. It has been suggested that the improved protein solubility after ultrasound treatment may result from opening and partial unfolding of the protein structure due to mechanical impact of the forces such as shear and microstreaming currents created by cavitation [45,46]. This may cause for example breakage of non-covalent interactions such as hydrophobic and electrostatic interactions and hydrogen bonding as well as improved interactions between protein and water molecules [22,23]. Moreover, the impact may be fortified by the impact of pH on protein structure during or after ultrasound treatment. However, contradictory data is available concerning impact of ultrasonication on the secondary structure of proteins. Ultrasound treatment in general has reduced the amount of alpha-helixes and increased the amount of beta-sheets for various plant proteins [19,21,23,46] but also some studies reported that it did not alter the secondary structure at all, but influenced more the tertiary structure [26,47]. As mentioned earlier, the impact on protein structure and functionality is varying depending on the ultrasonic intensity applied and the plant matrix.

#### 3.3.3. Combined Effect of pH-Shifting and Ultrasound Treatment

Shifting the pH of PEBF treated with ultrasound at pH 9 back to pH 7 resulted in a significantly higher protein solubility (40.7%) than what was observed for the control sample shifted to pH 9 and back to pH 7 without ultrasonication (26.1%). The solubility was also higher when compared to the control sample analysed at higher pH of 9 (37.3%) or the sample ultrasonicated at pH 7 (24.2%). Similarly, shifting the pH of BPI treated with ultrasound at pH 9 to pH 7 resulted in higher protein solubility (46.0%) when compared to the sample treated with ultrasound at pH 7 (25.6%) but the impact was less pronounced when compared to the control sample shifted to pH 9 and back to pH 7 (38.3%). Ultrasound treatment combined with pH shifting was previously found to increase the protein solubility of pea protein isolate from 10% (no ultrasound, only pH-shifting from 10 to 7) to 57% (ultrasonicated at pH 10 and shifted back to pH 7) [20]. Similarly, for soy proteins, the combined impact of pH-shifting to pH 9 and back to pH 7 followed by ultrasonication has increased the solubility from below 10% (only pH-shifting) to almost 80% (combined effect) [18]. Likewise, Yildiz et al. [16] showed that only pH-shifting of soy protein to pH 12 resulted in solubility of 57%, which was further improved to 83% by addition of mono-thermo-sonication during the pH-shifting phase. The observed increases in protein solubility propose the possibility to exploit ultrasound treatment combined with pH-shifting method as an effective tool to improve solubility of plant proteins that need to exhibit improved techno-functional properties at neutral pH-region. However, no data on the combined effect of ultrasonication and pH-shifting on improving cereal protein functionality has been earlier published but the current work demonstrates that similar improvements also apply for cereal proteins.

On the contrary to what was observed in the alkaline region, shifting the pH after ultrasonication at acidic conditions to neutral resulted in such a dramatic decrease in solubility of the ultrasound-treated BPI samples (from 69.7 to 11.2%) that even the impact of ultrasound treatment on improving the solubility became insignificant. For PEBF, shifting the pH of the ultrasound-treated sample from acidic conditions to neutral did not significantly alter the protein solubility (18.0% at pH 3 vs. 15.7% when shifted to pH 7). In accordance with these findings, Jiang et al. [20] observed only moderate increase in protein solubility after shifting pH from 2 to 7 after ultrasonication (18.3%) when compared to non-ultrasonicated sample shifted from pH 2 to 7 (9.6%). On the contrary, Lee et al. [18] combined pH-shifting to pH 2, 2.5 and 3 with ultrasonication and neutralisation, which resulted in increase in protein solubility from approximately 5% (only shifting to pH 2, 2.5 and 3, followed by neutralisation) to 65–75% when ultrasonication was included. This further suggests that the impact of ultrasound treatment varies depending on the plant matrix and protein isolation procedures applied during ingredient preparation.

### 3.4. Particle Size

**Figure 4:**
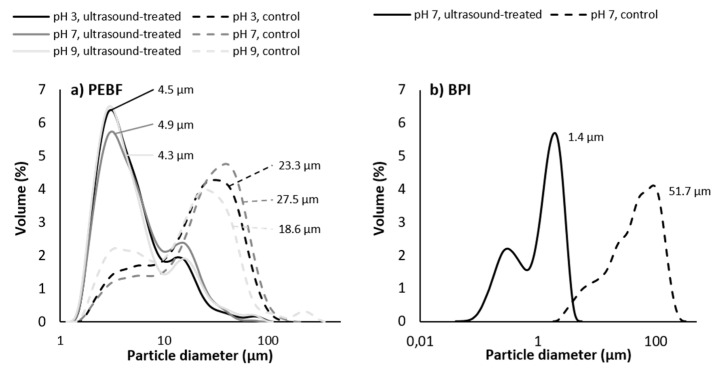
Impact of ultrasound treatment and pH on particle size distribution of (**a**) protein-enriched barley fraction (PEBF) and (**b**) barley protein isolate (BPI) at ultrasound-treatment pH. Median particle size of each sample is indicated next to the size distribution curve.

Particle size distribution of PEBF dispersion without ultrasound treatment was slightly affected by pH of the solution, and median particle sizes were 23.3, 27.5 and 18.6 µm for the dispersions at pH 3, pH 7 and pH 9, respectively (Figure 4a). This result is also in line with the initial protein solubility of the control samples, i.e., the sample with the highest median particle size exhibited the lowest protein solubility. Particle size distributions of the PEBF dispersions at all pH values were shifted towards smaller particles (Figure 4a) and median particle sizes were decreased to 4.5, 4.9 and 4.3 µm as a result of ultrasound treatment at pH 3, pH 7 and pH 9, respectively. Shifting pH to acidic or alkaline conditions prior to ultrasound treatment followed by adjustment to pH 7 after ultrasonication only had a minor impact on the particle size distribution (data not shown). Due to the presence of high amounts of starch in PEBF, the impact of ultrasound on particle size of this fraction presumably derived partly from detachment of the previously observed protein aggregates enclosing starch granules [7]. The larger peak represented the starch granules as well as the protein-starch aggregates present in the PEBF produced by size, density and shape-based separation of components in air classification, as previously discussed by Silventoinen et al. [7]. Analysis with light microscopy both with and without polarised light, however, revealed no changes in the structure or the size of the starch granules of the protein-enriched fraction (data not shown). Hence, impact of ultrasonication on starch in the PEBF should be examined in the following studies.

Particle size of BPI dispersion decreased enormously as a result of ultrasound treatment at pH 7 as the median particle size decreased from 51.7 to 1.4 µm (Figure 4b). As BPI sample was much more soluble than the less pure protein-enriched barley fraction and also the pH-shifting only had a minor effect on particle size, BPI samples were only analysed at neutral pH. Part of the large size of the isolate in control treatments was likely due to the isolation protocol applied to produce the sample. This may have caused formation of aggregates that were not detached during the laboratory type milling applied for this product after freeze drying. Ultrasound treatment efficiently reduced the size of such agglomerates.

### 3.5. Colloidal Stability

#### 3.5.1. Effect of pH-Shifting

**Figure 5:**
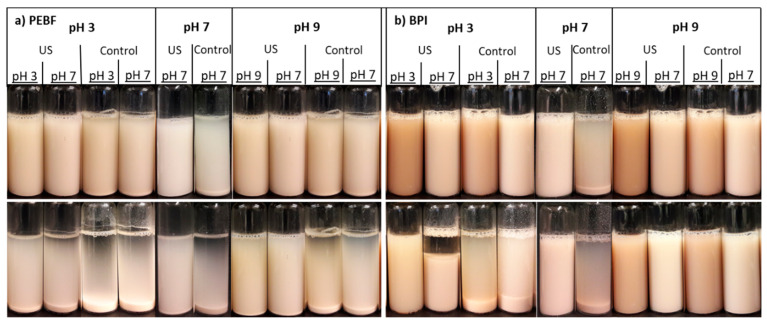
Impact of ultrasound treatment and pH on colloidal stability of (**a**) protein-enriched barley fraction (PEBF) and (**b**) barley protein isolate (BPI) dispersions at 1.5% (*w/w*) solid content after 10 min (upper row) and 180 min (lower row) of standing. The first text row indicates pH-value of the ultrasound/control treatment, the second row indicates whether the ultrasound treatment was performed for the sample or not, and the third row shows the colloidal stability analysis pH.

Stability of the samples against sedimentation without and after ultrasound treatment as well as after pH-shifting was studied by visual observations (Figure 5). Shifting of the pH alone had no clear impact on the colloidal stability of PEBF (Figure 5a). For BPI, shifting to neutral conditions from pH 3 (10 min time point) and especially from pH 9 (10 and 180 min time points) improved colloidal stability when compared to the control sample incubated at pH 7 (Figure 5b).

#### 3.5.2. Effect of Ultrasound Treatment

Ultrasound-treated PEBF exhibited improved stability at pH 3 after short (10 min) observation time when compared to the control sample at pH 3. However, the impact of ultrasound treatment was less visible after longer (180 min) time of standing. As no impact on protein solubility (Figure 3a) was observed after ultrasound treatment at pH 3 for PEBF, the slightly improved dispersion stability against sedimentation is not suggested to be due to effects of ultrasonication on protein solubility but due to the reduced particle size. At pH 7, the PEBF showed improved stability against sedimentation after ultrasound treatment, again in-line with observed reduction in particle size. At pH 9, the ultrasound treatment of PEBF improved both short- and long-term stabilities when compared to the non-ultrasonicated control. In that case, improved stability could be associated with improved protein solubility as well as with decreased particle size, as has also been discussed related to pea and soy protein isolates [25]. According to SDS-PAGE results, no proteolysis was observed, which indicated that the improved solubility and further improved colloidal stability resulted mainly from physical factors such as particle size reduction or structural changes such as possible modification of tertiary structure rather than protein hydrolysis. Decreasing particle size has been shown to improve stability of cereal-based ingredients in liquid matrices against gravitational sedimentation [37,48,49].

Dispersions of BPI were stable after short standing time (10 min) with and without ultrasound treatment at almost all of the studied pH values (Figure 5b). Only the control dispersion at pH 7 sedimented readily already after 10 min of standing. The result is in accordance with the low protein solubility of the control BPI at pH 7. During longer observation period, ultrasound treatment improved colloidal stability of the samples at pH 3 and pH 7. Both control and ultrasound-treated BPI dispersions were colloidally stable at pH 9 during the observation period.

Differences in the protein and starch contents between the PEBF and BPI were hypothesised to affect behaviour of the two raw materials in ultrasound treatment. However, the small differences between the contents of other components (20.4 and 13.5% in PEBF and BPI, respectively) were not supposed to cause major differences in the observed effects of ultrasound treatment. As majority of the BPI consisted of protein, protein solubility had more pronounced impact on the overall dispersion behaviour than for the PEBF that consisted mainly of starch. Thus, it is evident that the high protein solubility of BPI correlated more strongly with the increased colloidal stability than for PEBF where the insoluble starch contributed to the visually observed sedimentation over time. Impact of ultrasound on starch properties has also been a subject for several studies and significant effects were observed in destruction of granular structure [50], nanoparticle formation [51], and increased solubility, swelling power and water absorption capacity [52]. However, for example the nanoparticle formation enabling production of a transparent starch solution required extremely long treatment times (>75 min) [51] thus, in the present study it was not expected to obtain a similar impact on starch properties due to rather short treatment time.

#### 3.5.3. Combined Effect of pH-Shifting and Ultrasound Treatment

The ultrasound-treated PEBF shifted both from acidic (pH 3) and alkaline (pH 9) conditions to neutral exhibited improved colloidal stability after 10 min observation time when compared to the sample ultrasonicated at pH 7. On the contrary, for BPI, shifting from pH 3 to pH 7 after ultrasound treatment decreased the colloidal stability when compared to the sample that was ultrasonicated at pH 7. Similar impact was also observed as a decreased protein solubility. Both control and ultrasound-treated BPI dispersions that were shifted from pH 9 to neutral were colloidally stable during the observation period. Thus, it is evident that only pH-shifting to pH 9 was also able to improve the colloidal stability of BPI at pH 7.

In the current study, both pH-shifting and ultrasound treatment affected the protein solubility and dispersion stability properties of barley protein ingredients. The observed impacts of pH-shifting and ultrasonication on PEBF and BPI are proposed to result from the below-listed phenomena occurring during the treatments:

1) Mechanical impact by cavitation during ultrasonication resulting in reduction of particle size and therefore causing more interactions between protein and water and less sedimentation of protein, starch and their possible aggregates.
2) Partial protein unfolding during alkaline pH-shifting followed by partial refolding at netural pH and potential formation of a molten globule state with improved protein flexibility and properties.
3) Mechanical impact by cavitation during ultrasonication and pH shifting causing partial unfolding of the protein structure and possible modifications in tertiary structure, such as breakage of non-covalent hydrophobic and electrostatic interactions, and hydrogen bonding whereas primary structure remained unaffected.
4) However, in order to verify these hypotheses, further, more thorough, investigations are needed.

## 4. Conclusions

Ultrasound treatment of a protein-enriched barley fraction improved protein solubility and colloidal stability especially at alkaline pH. The impact was mainly due to the decrease in particle size as no impact on the primary protein structure was observed by SDS-PAGE analysis. For barley protein isolate, the ultrasound treatment improved protein solubility the most pronouncedly at acidic (pH 3) and alkaline (pH 9) conditions whereas lesser impact was seen at neutral pH 7. Similarly, improved colloidal stability was observed at all the studied pH-values. In addition to the impact of ultrasound treatment, effect of pH-shifting on the properties of the two barley ingredients was elucidated. Shifting from alkaline to neutral pH after ultrasound treatment retained the improved colloidal stability when compared to samples treated with ultrasound directly at neutral pH. This suggests possible use of ultrasound as a pre-treatment for improving applicability of barley protein ingredients in liquid food systems (e.g., non-dairy milk substitutes) at neutral pH. Potential effect of pH-shifting and ultrasound treatment on other functional properties of barley proteins relying on protein solubility, such as gelation, emulsification and foaming, should be investigated in the following studies.

## Figures and Tables

**Table 1:** Protein and starch content in the protein-enriched barley fraction (PEBF) and barley protein isolate (BPI).

	Protein-Enriched Barley Fraction (PEBF)	Barley Protein Isolate (BPI)
Protein (% dm) ^a^	24.0 ± 0.20	85.9 ± 0.43
Starch (% dm) ^a^	55.6 ± 0.01	0.6 ± 0.02
Other (% dm)	20.4	13.5

^a^ ± average deviation.
